# Growth Hormone Alters Remapping in the Hippocampal Area CA1 in a Novel Environment

**DOI:** 10.1523/ENEURO.0237-24.2024

**Published:** 2025-02-11

**Authors:** Kamilla G. Haugland, Sondre Valentin Jordbræk, Erik Knutsen, Kirsten B. Kjelstrup, Vegard H. Brun

**Affiliations:** ^1^Departments of Clinical Medicine, UiT – The Arctic University of Norway, Tromsø 9019, Norway; ^2^Medical Biology, UiT – The Arctic University of Norway, Tromsø 9019, Norway; ^3^University Hospital of North Norway, Tromsø 9019, Norway

**Keywords:** endocrine, growth hormone, hippocampus, memory, place cell

## Abstract

Growth hormone (GH) is a neuromodulator that binds to receptors in the hippocampus and alters synaptic plasticity. A decline in GH levels is associated with normal aging, stress, and disease, and the mechanisms proposed involve the hippocampal circuit plasticity. To see how GH affects the hippocampal neural code, we recorded single neurons in the CA1 region of male Long–Evans rats with locally altered GH levels. Rats received injections of adeno-associated viruses into the hippocampus to make the cells overexpress either GH or an antagonizing mutated GH (aGH). Place cells were recorded in both familiar and novel environments to allow the assessment of pattern separation in the neural representations termed remapping. All the animals showed intact and stable place fields in the familiar environment. In the novel environment, aGH transfection increased the average firing rate, peak rate, and information density of the CA1 place fields. The tendency of global remapping increased in the GH animals compared with the controls, and only place cells of control animals showed significant rate remapping. Our results suggest that GH increases hippocampal sensitivity to novel information. Our findings show that GH is a significant neuromodulator in the hippocampus affecting how place cells represent the environment. These results could help us to understand the mechanisms behind memory impairments in GH deficiency as well as in normal aging.

## Significance Statement

Early in life, brain plasticity is particularly useful for adaptation to the environment and encoding of new memories. Once useful strategies are learned, stabilizing existing neural representations could be more beneficial. To understand how the brain's degree of plasticity can be altered, we have studied if growth hormone (GH) influences how principal cells in the hippocampus respond to changes in the environment. GH is a relevant neuromodulator because of its declining level with age and because of its secretion during memory consolidation in sleep. Understanding how memory and brain plasticity are affected by hormones could help us understand both normal cognitive changes throughout life and memory problems involved in aging or hormone deficiency.

## Introduction

The hippocampus has an enormous storage capacity with neural activity that represents previous experiences (Shapiro, 2001; Moser et al., 2015; Eichenbaum, 2017; Lisman et al., 2017; Colgin, 2020). The place cells of the hippocampus fire in a location-specific manner (O'Keefe and Dostrovsky, 1971), representing both spatial information (place field) and the current configuration of sensory input and internal state (firing rate). As the firing patterns of the place cells are flexible (Cobar et al., 2017), the cell activity can easily be adjusted to changes in the animal's experience (Cohen et al., 2017). A common approach to investigate the plasticity of place cells is therefore to record the neural activity when the animal is exploring different environments (Muller and Kubie, 1987; Thompson and Best, 1989; Leutgeb et al., 2004, 2005; Alme et al., 2014). Place cells with a particular firing pattern in one environment may change the firing properties when the animal explores another environment or when significant parts of the environment are exchanged, a phenomenon known as remapping (Colgin et al., 2008; Latuske et al., 2017). The type of remapping with the greatest change in neural activity is referred to as global remapping (Muller and Kubie, 1987; Leutgeb et al., 2004, 2005; O'Neill et al., 2008). In global remapping, the cell ensembles reorganize orthogonally, and such events are commonly observed when the animal visits a novel room. Another version of remapping is rate remapping, in which the place fields remain in the same location, but the firing rates change (Wood et al., 2000; Ainge et al., 2012; Allen et al., 2012). This rate modulation is suggested to depend on nonspatial variables and is apparent with more subtle spatial changes, like when the animal explores a novel apparatus in a familiar room. Despite more pronounced changes in firing rate in CA3 after changes in the shape of apparatus, CA1 also exhibits rate differences under similar conditions (Leutgeb et al., 2005) which are associated with task demands (Sanders et al., 2019).

Although studies have described various types of remapping events as direct responses to changes in sensory information, remapping may also be dependent on the intrinsic plasticity of the hippocampus (Cobar et al., 2017). In such a way, the plasticity of place cells could reflect awareness of changes in the environment. The activity of the place cells is especially sensitive to local modulation by neuromodulators (Kentros et al., 2004; Tran et al., 2008; Teles-Grilo Ruivo and Mellor, 2013; Brandon et al., 2014; Atherton et al., 2015). Neuromodulators primarily alter the dynamics of excitatory and inhibitory synaptic transmission, modifying synaptic processes and changing the resting membrane potential (Edelmann et al., 2017; Palacios-Filardo and Mellor, 2019).

Growth hormone (GH) is a local neuropeptide expressed in the hippocampus with the ability to increase cognitive performance (Aberg et al., 2006; Nyberg and Hallberg, 2013; Ashpole et al., 2015; Martin-Rodriguez et al., 2020). We and others have previously shown that GH improves spatial memory and learning processes (Li et al., 2011; Haugland et al., 2020), as well as enhancing hippocampal spine density (Haugland et al., 2020; Nylander et al., 2020). Studies also show that GH enhances hippocampal synaptic potential and spatial cognition after sleep deprivation (Kim et al., 2010; Arvin et al., 2023). However, it remains unknown whether this increase in hippocampal plasticity affects the information processes displayed by changes in place cell firing. By using recombinant adeno-associated viruses (rAAVs), we can overexpress GH or an antagonist of GH receptor (aGH) to locally manipulate the levels of GH in the hippocampus. In this study, we investigate how GH or aGH affects hippocampal place cell firing when the animal explores a novel environment.

## Materials and Methods

### Subjects

In total, 23 male Long–Evans rats (Charles River Laboratories) were housed in single cages after implantation, in a humidity- and temperature-controlled environment. We used only male animals to control for changes in the estrous cycle that could mask or confound the effects of GH, because GH secretion differs between males and females. All animals were kept at 90–100% of free-feeding body weight and maintained on a 12 h light/dark schedule. Twelve animals were used for single-unit recordings while 11 were used for qRT-PCR. Single-unit recordings were conducted in the dark phase. The experiments were performed in accordance with the Norwegian Animal Welfare Act and the European Convention for the Protection of Vertebrate Animals Used for Experimental and Other Scientific Purposes.

### Viruses

We provide a brief description of the viruses used in this study as this method is previously described in detail (Haugland et al., 2020). Three recombinant adeno-associated viruses (rAAVs) 1/2 chimeric pseudotypes (kindly provided by Ki Ann Goosens, Massachusetts Institute of Technology) were used to either overexpress GH and green fluorescent protein (GFP), mutated antagonizing GH (aGH) and GFP, or GFP only. The expression sequences were rAAV-CMV-GH-IRES-GFP, rAAV-CMV-aGH-IRES-GFP, or rAAV-CMV-IRES-GFP. Animals were randomly assigned to receive the different viruses, and successful single-unit recordings were made in 12 animals (GH, *n* = 3; aGH, *n* = 4; control, *n* = 5), while 11 animals were used for qRT-PCR (GH, *n* = 4; aGH, *n* = 4; control, *n* = 3). The rAAV-GFP-GH was constructed with the *Gh* gene (GeneBank Accession Number U62779.1). The rAAV-GFP-aGH contained a *Gh* gene with a single amino acid substitution (rGH-G144R) to produce a mutant GH protein with antagonist activity on the GH receptor. This mutation is equivalent to human G120R and therefore also described as the 120R mutation of the GH.

### Stereotaxic surgery

The rats (350–400 g at surgery) were anesthetized in an induction chamber with isoflurane for surgery and received subcutaneous buprenorphine and meloxicam to minimize postoperative discomfort. When fully anesthetized, the skull was fixed in a Kopf stereotaxic frame. The depth of anesthesia was monitored by heart rate and oxygen saturation (Kent Scientific), as well as clinically by regular reflex testing and breathing monitoring. The local anesthetic bupivacaine was given subcutaneously, and the skin was disinfected with 70% ethanol before incision. Holes in the skull were drilled at appropriate locations on each hemisphere. The viruses were injected with a sterile 2 µl Hamilton syringe (Hamilton Company). The animals received four injections of 0.4 µl virus solution (0.2 µl/min) in each hemisphere in the dorsal hippocampus according to the injection coordinates calculated anteroposteriorly (AP), mediolaterally (ML) from bregma, and dorsoventrally (DV) from dura: AP −3.0 mm, ML ±1.2 mm, DV 2.3 mm; AP −3.5 mm, ML ±2.2 mm, DV 2.2 mm; AP −4.0 mm, ML ±2.4 mm, DV 2.4 mm; and AP −4.4 mm, ML ±3.5 mm, DV 2.8 mm. The needle was left at the site of injection for 5 min after each injection to allow diffusion of the virus before slow retraction. After the virus injections, one or two microdrives were implanted above hippocampal CA1 (AP −3.5, ML ±3.3, DV −1.5), each with a bundle of four tetrodes. The implant was secured to the skull with dental cement and Histoacryl, and screws were attached to the skull. After surgery, the animals recovered in their home cage and were closely monitored for a minimum of 3 days postsurgery.

### Recording procedures

The local field potentials (LFP) were recorded using tetrodes attached to microdives (Axona Ltd, UK). Tetrodes were constructed from four twisted 17 μm polyamide-coated platinum–iridium (90–10%) wires (California Fine Wire). Before implantation, the electrode tips were plated with platinum to reduce electrode impedance to between 120 and 220 kΩ at 1 kHz.

Recordings of LFP started 1 week after implantation of the tetrodes. Rats were connected to an Axona data acquisition system via a wire connecting the microdrive to a preamplifier to digitize the signals at 24-bit resolution and 48 kHz sampling rate. The signals were amplified by a system unit which also contained a video tracker. The weight of the cable to the preamp was counterbalanced by wires attached to the ceiling with a counterweight. The unit activity was amplified between 4,000 and 14,000 times and high-pass filtered with 360 Hz cutoff and low-pass filtered at 7 kHz. For the EEG, the signals were bandpassed with a low-pass filter with 500 Hz cutoff with a 4,800 Hz resolution. An overhead camera recorded the position of the light-emitting diode (LED) on the head stage of the microdrive. The tetrodes were lowered daily in steps of 50 μm between sessions until the hippocampus was reached. Recording started when the tetrodes were in CA1 with putative principal cells and theta modulation was present in the EEG.

### Behavioral procedures

After recovering from the surgeries, the rats were habituated to a square arena (100 cm × 100 cm with 50-cm-high walls) to become a familiar to the environment (Fig. 2*A*). A cue card centered on one of the walls was used as a local cue. Dark curtains covered parts of the recording arena with distance cues. The animals were trained daily to forage for crumbs of cookies. Before a trial session in the box, the animal rested on a pedestal in a small pot with a blanket and a warm heating bottle (named pot sessions). After trials, the animals were allowed back to the pot. The apparatus was cleaned between each trial. When the animals were familiar with the box (>5 d of experience) and place cells were well separable during offline analysis, the rats were introduced to a novel environment consisting of a novel circular apparatus in the same room, with the same floor and distance cues.

The standard trial sequence started with a pot session (5 min), followed by two sessions in the familiar box (10 min) separated by 10 min, before the last pot session (5 min). When a sufficient number of single units were separable, the trial sequence included a novel circle (10 min) in between the familiar box trials. The circle arena remained novel during the experiments as each animal only visited the circle 1–3 times. Each trial was typically separated by a few minutes to allow cleaning of the arenas and clustering and inspection of recorded cells by the experimenter.

### Spike sorting and place fields

Spike sorting was performed offline using graphical cluster-cutting software (TINT, Axona Ltd). The spikes were manually clustered in 2D projections of the multidimensional parameter space (consisting of waveform amplitudes and waveform energy), using autocorrelation and cross-correlation functions as additional separation criteria. The cluster separation was assessed by calculating the distance between spikes of different cells in Mahalanobis space (isolation distance). Clusters that remained stable across recording trials were regarded as the same unit. Putative excitatory cells were distinguished from putative interneurons based on the width of the extracellular action potential, firing pattern (complex spikes), and average rate. Only active place cells with an average firing rate of a minimum of 0.25 Hz in at least one of the sessions were included in the analysis.

To characterize place fields, a spike density function was estimated by convolving the spike train (sum of Dirac delta functions) with a smoothing kernel (2 s Blackman window, normalized to unit gain at zero frequency). The spike density function was sampled synchronously with a position tracker. The rate map was calculated as a weighted mean of the sampled spike density function, with weights given by Euclidian distance and a 30-cm-wide Blackman window. The rate map was calculated for pixels of 5 cm × 5 cm visited by the rat. Field size was defined as the size of the largest cluster of neighboring pixels with a firing rate above 20% of the peak rate. The percentage of bursting was calculated as the number of bursts divided by the number of bursts plus the number of single spikes. The spatial information (bits/spike) was calculated as follows:

$$I=\overset{N}{\underset{i=1}{\sum }}\;p_{i}\frac{\lambda_{i}}{\lambda }\log_{2}\frac{\lambda_{i}}{\lambda },$$

where *I* is the information density, *λ_i_* is the mean firing rate in the *i*-th bin, and *λ* is the overall mean firing rate. *p_i_* is the probability of the animal being in the *i*-th bin, as previously described (Skaggs et al., 1996). The sparseness (*S*) was calculated by dividing the square mean rate (*λ*^2^) by the mean square rate over all pixels (Skaggs et al., 1996):

$$S=\frac{\lambda^{2}}{\sum_{i}^{N}\;p_{i}{\left(\lambda_{i}\right)}^{2}},$$

Spatial correlation was estimated as the first-order spatial autocorrelation of the place field maps between sessions and measured the extent to which the firing rate in a pixel is predicted by the rates of the neighboring pixels.

The rate overlap between two recording trials was estimated by dividing, for each cell, the average firing rate in the less active enclosure by the average firing rate in the more active arena. The ratios were averaged for all the cells active in the arenas. The resulting overlap scores would be equal to 1 if the average firing rates were equal, while the score would be toward 0 if the cell was only active in one environment. The rate change was calculated by the absolute change in average firing rate between the environments.

### Sharp wave ripple detection and analysis

Signals were downsampled to a rate of 1,200. The signals were filtered between 90 and 250 Hz and then squared and normalized. A wavelet transform method was used to compute a high resolution of the time-varying power (*V*^2^) of different frequency bands (BuzCode function bz_WaveSpec.m from the MATLAB BuzCode; https://github.com/buzsakilab/buzcode). The time–frequency analysis was achieved by creating a family of complex Morlet wavelets convoluted with the data via the fast Fourier transform. The family of wavelets was characterized by a constant wavelet of seven cycles. Sharp wave ripples (SWRs) can be detected in the CA1 stratum radiatum as large amplitude negative polarity deflections and are often associated with short oscillatory patterns of LFP in the CA1 pyramidal layer, commonly known as “ripples” (Buzsaki, 2015). In our study, SWRs were detected using bz_findRipples.m from the MATLAB BuzCode, using a 30 ms merge time, minimum duration of 15 ms, maximum duration of 200 ms, detection threshold of three standard deviations (SD), and peak threshold of five SD. To reduce contamination from high-gamma events, candidate ripples with a peak frequency of <115 Hz were eliminated. The speed was computed from position data, and only SWRs during immobility were used for the analysis (animals moved ≤5 cm/s).

SWR rate was calculated by dividing all ripples by the detected time of immobility. The peak power was found using time–frequency analysis on each ripple and detecting the frequency with the highest power. Sessions with <10 ripples were excluded from the analysis.

### Theta-power and gamma detection

Theta and gamma oscillations were collected by time–frequency analysis of regions, and activity over three times mean ± SD power was summed up. Theta cycles were detected using a bandpass filter between 4 and 12 Hz. For slow gamma and fast gamma, filters of 25–50 Hz and 65–140 Hz were used, respectively, and normalized.

### Histology

The rats were deeply anesthetized with isoflurane gas and given buprenorphine (0.05 mg/kg) and a lethal dose of pentobarbital (100 mg/kg), before being perfused intracardially with PBS and 4% formaldehyde at 80 ml/min using a peristaltic pump (World Precision Instruments). The brains were extracted and stored in 4% formaldehyde at 4°C. Before sectioning, the brains were quickly frozen and placed on a cryostat. In addition, 40 μm coronal sections were cut on Leica CM1950 cryostat (Leica Biosystems) and mounted on superfrost object glasses. The tetrode traces and the viral expression were detected in the fluorescence microscope using Axio Zoom V.16 (Carl Zeiss).

### RNA isolation, cDNA synthesis, and qRT-PCR

The dissected brains were homogenized in 1 ml of TRI Reagent (Zymo Research) using the Precellys tissue homogenizers [MagNA Lyser Green Beads with ceramic beads (Roche), 6,000 rpm, 1 × 20 s]. Total RNA was isolated from the homogenized brains using the Direct-zol RNA MiniPrep (Zymo Research) kit according to the manufacturer's recommendations. RNA concentration was measured by Qubit (Thermo Fisher Scientific). DNA contamination was removed using 0.5U DNase I (Ambion) following heat inactivation. cDNA synthesis of DNase-treated total RNA was performed with SuperScript IV Reverse Transcriptase (Thermo Fisher Scientific). In addition, 2.5 μM of random hexamer primer (Thermo Fisher Scientific) and 500 ng of template were used for the reaction (10 μl total reaction volume). RNA was denatured at 65°C for 5 min, and cDNA was synthesized at 53°C for 10 min. cDNA was diluted 1:10 in nuclease-free H_2_O.

For qRT-PCR, 2.5 μl cDNA was mixed with 5.0 μl FastStart Essential DNA Green Master (Roche Life Science) and 2.5 μl forward and reverse primer mix (1 μM each). All primer sequences are provided in Table 1. The LightCycler 96 was used for quantification, and the expression of *Gh* is presented as 2^−ΔCq^ using the geometric mean of *Hprt*, *B2m*, and *Actb* as internal reference.

**Table 1.** Primer sequences

Gene	Forward primer	Reverse primer
*Hprt*	AGCAGACGTTCTAGTCCTGTG	CAAAAGGGACGCAGCAACAG
*B2m*	ACTGAATTCACACCCACCGA	TACATGTCTCGGTCCCAGGT
*Actb*	GCAGATGTGGATCAGCAAGC	GCAGCTCAGTAACAGTCCGC
*Gh1*	AATTGCTTCGCTTCTCGCTG	AGTTTCTCATAGACGCGGTCC

Primer sequences for qRT-PCR for the genes *Hprt*, *B2m*, *Actb*, and *Gh1*.

### Sanger sequencing

For verification of expression of wild-type and antagonist *Gh* mRNA, Sanger sequencing across the mutated region of *Gh* was performed. One microliter of cDNA was used as input in a 20 μl PCR reaction, using the PrimeSTAR GXL DNA Polymerase (Takara) according to the manufacturer's recommendations. PCR primers used were as follows: GAC AAA GTG TAG GGG TGG CA (forward) and TTC ATG ACC CGC AGG TAG GT (reverse). PCR reactions were run on a 1% agarose gel, and the PCR bands at 510 nt were cut out of the gel and purified using the QIAquick Gel Extraction Kit (Qiagen). Samples were sequenced at the DNA sequencing core facility UNN using the Applied Biosystems 3130xl Genetic Analyzers. For sequencing, the forward PCR primers were used.

### Experimental design and statistical analysis

The results are presented as mean ± SEM and were tested for normal distribution using Shapiro–Wilk normality test. If the data passed the normality test, parametric tests (ANOVA and *t* test) were used for statistical analysis, while the nonparametric tests (Kruskal–Wallis test and Mann–Whitney test) were used when the data were not normalized. The place cell firing was analyzed using MATLAB (MathWorks), and statistical analysis and graphs were made using GraphPad Prism version 7.09 (GraphPad Software) with *α* = 0.05.

## Results

**Figure 1.**
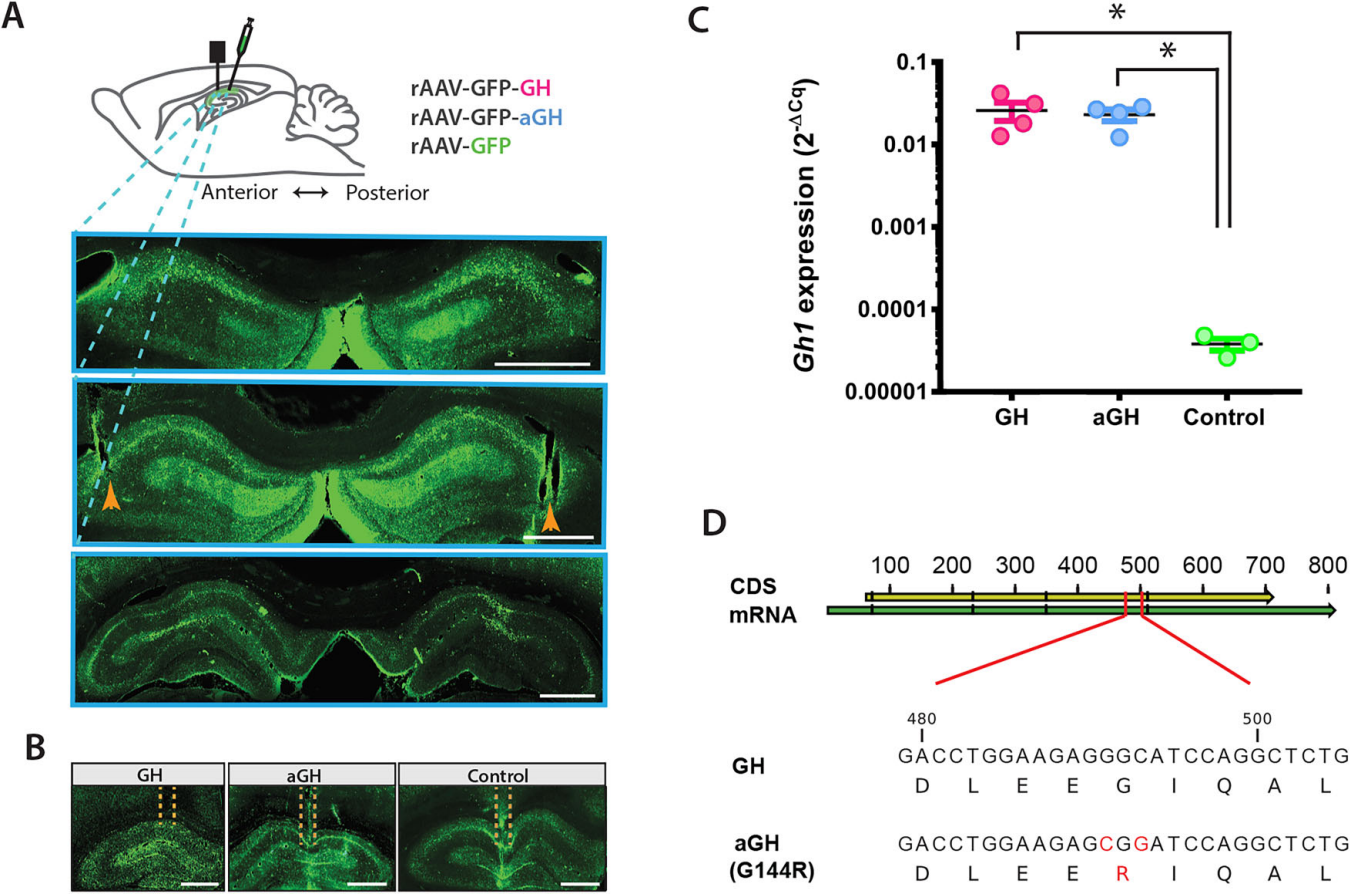
Overexpression of growth hormone or antagonizing growth hormone in the dorsal hippocampus. ***A***, Top panel, Sagittal view of the rat brain illustrating the viral injections by recombinant adeno-associated viruses (rAAVs) to provide overexpression of growth hormone (GH) or antagonizing GH (aGH). The tetrodes were placed in between viral injections in the dorsal hippocampus. Bottom panel, Three coronal sections of the dorsal hippocampus with blue dotted lines showing the anteroposterior positions. The orange arrowhead points at the tetrode traces in each hippocampus. The green fluorescent protein (GFP) in green indicates viral transfection. Scale bar, 1,000 μm. ***B***, Tetrode traces and GFP expression in representatives for each group, the GH, aGH, and the control, respectively. Scale bar, 1,000 μm. ***C***, qRT-PCR for growth hormone 1 (*Gh1*) showed sensitivity to both the GH and aGH gene expressions (one-way ANOVA *F*_(2,8)_ = 7.774, *p* = 0.0113; unpaired two-tailed *t* test GH control, *p* = 0.0205; unpaired two-tailed *t* test aGH control, *p* = 0.0034). ***D***, Sequencing the coding sequence (CDS) from the viral transfected tissues, we verified the G114R mutation in the aGH samples only, and not in the GH.

A total of 23 rats were used in this study, of which 12 received both viral injections and microdrive implantations. The histology revealed that the tetrodes were lowered into the dorsal CA1 among transfected cells, shown by the expression of GFP (Fig. 1*A*,*B*). The overall *Gh* mRNA in the animals was quantified by qRT-PCR, which revealed significantly increased levels of *Gh* in the GH and aGH group compared with controls (Fig. 1*C*; one-way ANOVA *F*_(2,8)_ = 7.774, *p* = 0.0113; unpaired two-tailed *t* test GH control, *p* = 0.0205; unpaired two-tailed *t* test aGH control, *p* = 0.0034). Since the qRT-PCR test was sensitive to both the GH and aGH mRNA's, we further sequenced the mRNAs of GH versus aGH (Fig. 1*D*) to verify the G144R mutation in the aGH gene that prevents the activation of GH receptor after binding of the aGH ligand.

### Increased place cell firing after overexpression of growth hormone antagonist in a familiar environment

We examined the hippocampal place cell activity by recording cells in different environments, including a familiar box and a novel circle, in the same room. A total 224 place cells were included in the following analyses (control, *n* = 71; aGH, *n* = 124; GH, *n* = 31).

**Figure 2.**
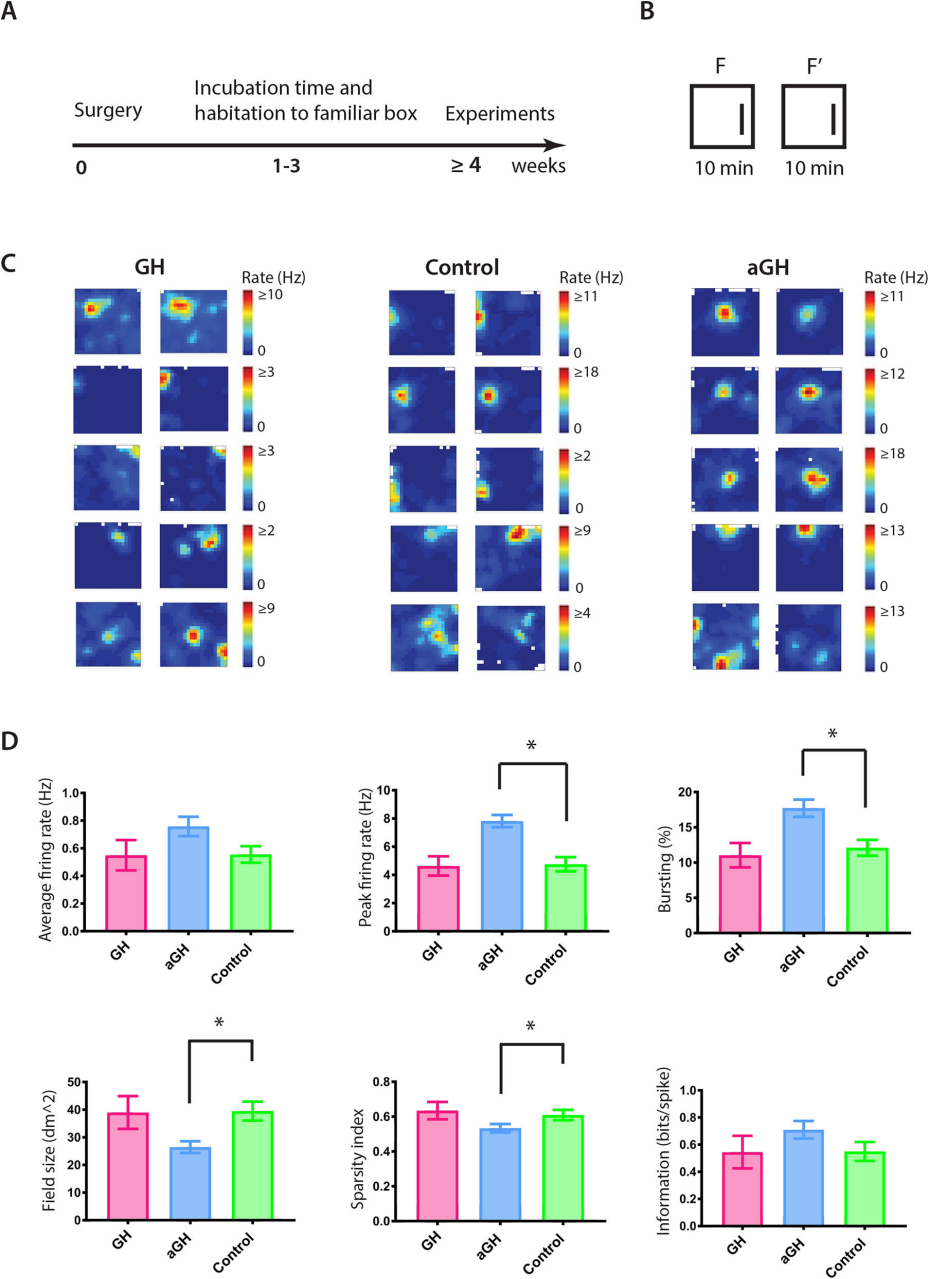
Growth hormone antagonist increases the place cell firing activity in a familiar environment. ***A***, Timeline of the experiment. Rats received surgeries with simultaneous viral injections and electrode implantation. After a recovery period of 1 week, the animals were habituated to a square box (1 × 1 m) with a local cue. Three weeks of incubation time for viral transfections were allowed before the animals were subjected to single-unit recordings. ***B***, For experiments in the familiar environment, sequential recordings in a familiar box were separated by a 10 min break, to check for stable place fields in the hippocampal CA1 region. ***C***, Representative place cells with their distinct place fields are shown for each of the groups. Color bars indicate firing rates, with red illustrating the maximum firing location of the cells (place fields) and dark blue indicating firing rates (hertz) toward zero. All the groups display place cells with stable place fields in the first (F) and the second (F’) recording in the familiar environment. ***D***, Firing properties of the place cells. The average firing rate (hertz) in the familiar environment did not differ between the groups (Kruskal–Wallis test, *p* = 0.7095). However, the peak rate (hertz) was significantly higher in the aGH group (Kruskal–Wallis test, *p* < 0.0001; Mann–Whitney test aGH control, *p* < 0.0001) compared with the control. aGH increased bursting (Kruskal–Wallis test, *p* = 0.0012; Mann–Whitney test aGH control, *p* = 0.0014) but decreased the field size (square decimeter; Kruskal–Wallis test, *p* = 0.0022; Mann–Whitney test aGH control, *p* = 0.0024) and the sparseness (Kruskal–Wallis test, *p* = 0.0494; Mann–Whitney test aGH control, *p* = 0.0455). The information score did not differ between the groups (Kruskal–Wallis test, *p* = 0.1412).

**Figure 3.**
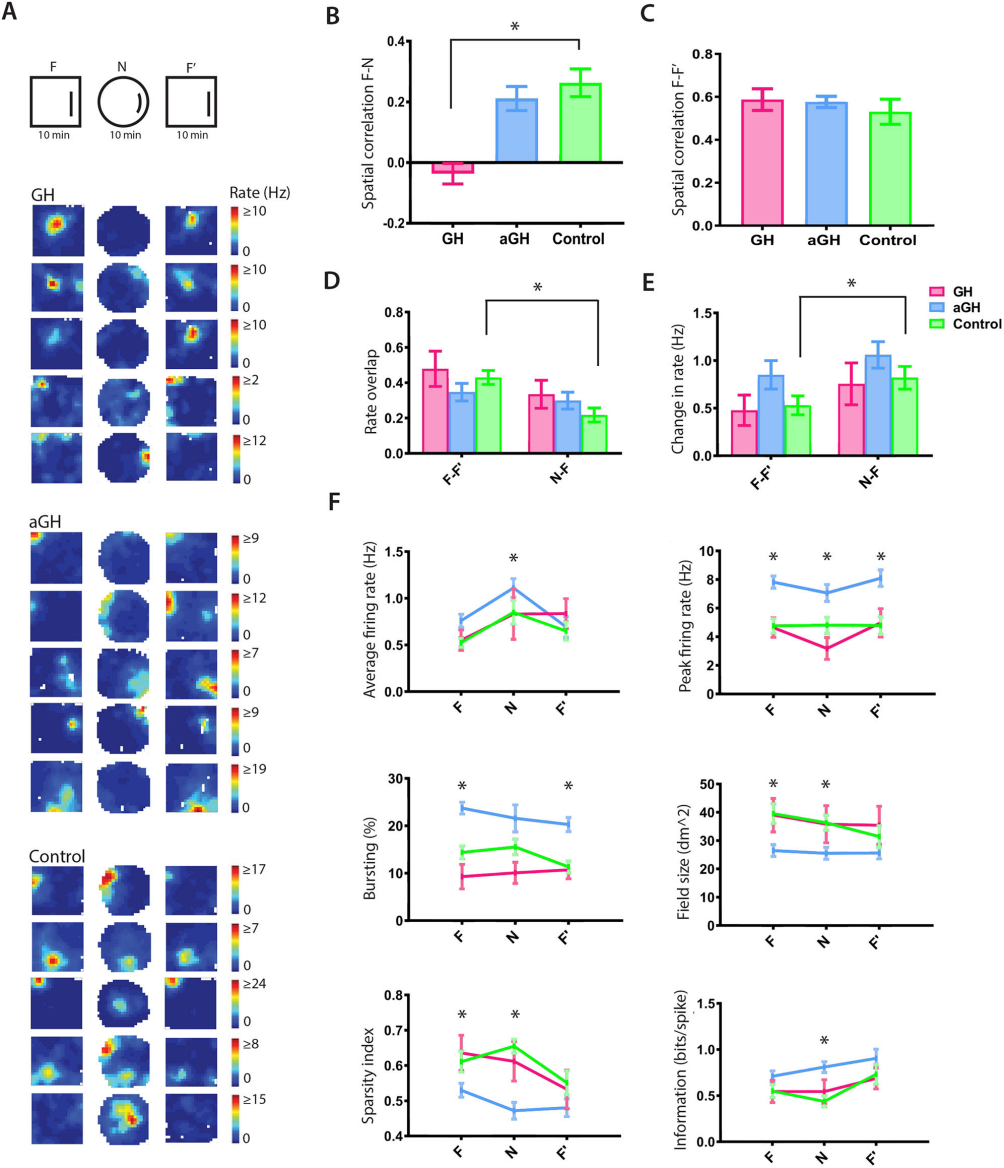
Growth hormone modulates remapping in the hippocampus. ***A***, Top panel, Experimental design consisting of the familiar environment and a novel environment. The animals were first allowed to explore the familiar environment (F) before visiting a novel environment (N) and then put back to the familiar environment (F’). Bottom panel, Representative place cells for each group with the maximum firing rate (hertz) shown in red and minimum firing rate shown in blue. In the GH animals, place fields changed their spatial location between the familiar and novel environments, while place cells of the aGH and control groups tended to keep their place fields in the novel environment. ***B***, Spatial correlation analysis was used to investigate whether the place fields remain stable between the familiar and novel environments or if they had changed locations. We found that GH significantly reduced the spatial correlation (Kruskal–Wallis test, *p* = 0.0055; Mann–Whitney test aGH control, *p* = 0.0012) as compared with controls. The GH group is illustrated in pink, aGH in blue, and control in green. ***C***, The spatial correlation between the familiar environments (F and F’) is similar for all the groups (Kruskal–Wallis test, *p* = 0.9195). ***D***, We observed significant changes in the control group regarding the rate overlap between the familiar and novel environments (Mann–Whitney test aGH control, *p* = 0.0258). ***E***, Also, the change in firing rate (hertz) between the familiar and novel environments was only seen in the control group (Mann–Whitney test aGH control, *p* = 0.0359). ***F***, Firing properties of place cells. aGH increased the firing rate (hertz) in the novel environment (Kruskal–Wallis test, *p* = 0.0194; Mann–Whitney test aGH control, *p* = 0.0146). Cells from aGH animals had higher peak firing rates (hertz) throughout the experiments, including the familiar and novel environments (Kruskal–Wallis test in N, *p* < 0.0001; Mann–Whitney test aGH control in N, *p* = 0.0007; Kruskal–Wallis test in F’, *p* < 0.0001; Mann–Whitney test aGH control in F’, *p* < 0.0001) and increased bursting (%) in both the familiar environments (Kruskal–Wallis test in F’, *p* < 0.0001; Mann–Whitney test aGH control in F’, *p* < 0.0001) although it was not a significant change in the novel environment (Kruskal–Wallis test in N, *p* = 0.0529). In the novel environment, the field size (square decimeter) and the sparseness decreased in the aGH group (field size: Kruskal–Wallis test in N, *p* = 0.0098; Mann–Whitney test aGH control in N, *p* = 0.0014; Sparsity: Kruskal–Wallis test in N, *p* < 0.0001; Mann–Whitney test aGH control in N, *p* < 0.0001), while the information (bits/spike) increased (Kruskal–Wallis test in N, *p* < 0.0001; Mann–Whitney test aGH control in N, *p* < 0.0001).

We first investigated the impact of GH modulations on place cells in a familiar environment. Recordings in the familiar environment were divided into two 10 min sessions separated by 10 min (Fig. 2*B*). All of the groups displayed place cells that were active in the familiar environments (Fig. 2*C*). Spatial correlation confirmed stable place fields across the familiar box trials (Fig. 3*C*; GH, *r* = 0.58; aGH, *r* = 0.58; control, *r* = 0.53; Kruskal–Wallis test, *p* = 0.9195). The aGH significantly impacted the firing properties of the place cells (Fig. 2*D*). Although the average firing rate did not differ between the groups (Kruskal–Wallis test, *p* = 0.7095), the aGH increased the peak rate (Kruskal–Wallis test, *p* < 0.0001; Mann–Whitney test aGH control, *p* < 0.0001) and the bursting (Kruskal–Wallis test, *p* = 0.0012; Mann–Whitney test aGH control, *p* = 0.0014). In addition, place cells of the aGH animals displayed smaller field size (Kruskal–Wallis test, *p* = 0.0494; Mann–Whitney test aGH control, *p* = 0.04555) and a reduction in the place field sparseness (Kruskal–Wallis test, *p* = 0.0022; Mann–Whitney test aGH control, *p* = 0.0024) as compared with controls.

### Growth hormone affects place cell representations in a novel environment

Since GH alters hippocampal plasticity (Kim et al., 2010; Molina et al., 2012, 2013; Haugland et al., 2020), we wanted to investigate if GH could modulate the place cell activity during the first experience in a novel environment (Fig. 3*A*). After exploration in the familiar environment (F), the rats were introduced to a novel circular environment in the same room (N), before returning to the familiar environment (F’). Interestingly, the place cells of the GH animals remapped more than control animals (Fig. 3*B*; Kruskal–Wallis test, *p* = 0.0055; Mann–Whitney test, *p* = 0.0012), and none of the GH cells had a positive spatial correlation between F and N, with an average spatial correlation of *r* = −0.036. aGH and controls had a weak spatial correlation (aGH, *r* = 0.211; control, *r* = 0.263). Place cells remained stable between the two sessions in the familiar environment and did not differ between the groups (Fig. 3*C*).

Only the control group displayed a significant difference in rate overlap (Fig. 3*D*) and rate change (Fig. 3*D*,*E*; Mann–Whitney test, *p* = 0.0258; Mann–Whitney test, *p* = 0.0359) when comparing the respective values in the familiar environment sessions (F–F’) and the familiar-to-novel environment (F–N). The changes observed in rate overlap and rate change indicate that only controls displayed rate remapping when introduced to the novel environment.

In the novel environment, aGH maintained the high firing rates from the familiar environment (Fig. 3*F*). The peak firing rate of the aGH animals was high throughout the experiments, including both the familiar environment sessions and the novel environment (Kruskal–Wallis test in N, *p* < 0.0001; Mann–Whitney test aGH control, *p* = 0.0007; Kruskal–Wallis test in F’, *p* < 0.0001; Mann–Whitney test aGH control in F’, *p* < 0.0001). In the novel environment, aGH also increased the average firing rate (Kruskal–Wallis test, *p* = 0.0194; Mann–Whitney test, *p* = 0.0146) and the information (Kruskal–Wallis test, *p* < 0.0001; Mann–Whitney test, *p* < 0.0001), while the field size and sparseness were significantly reduced (field size: Kruskal–Wallis test in N, *p* = 0.0098; Mann–Whitney test aGH control in N, *p* = 0.0014; sparseness: Kruskal–Wallis test, *p* < 0.0001; Mann–Whitney test, *p* < 0.0001).

**Figure 4.**
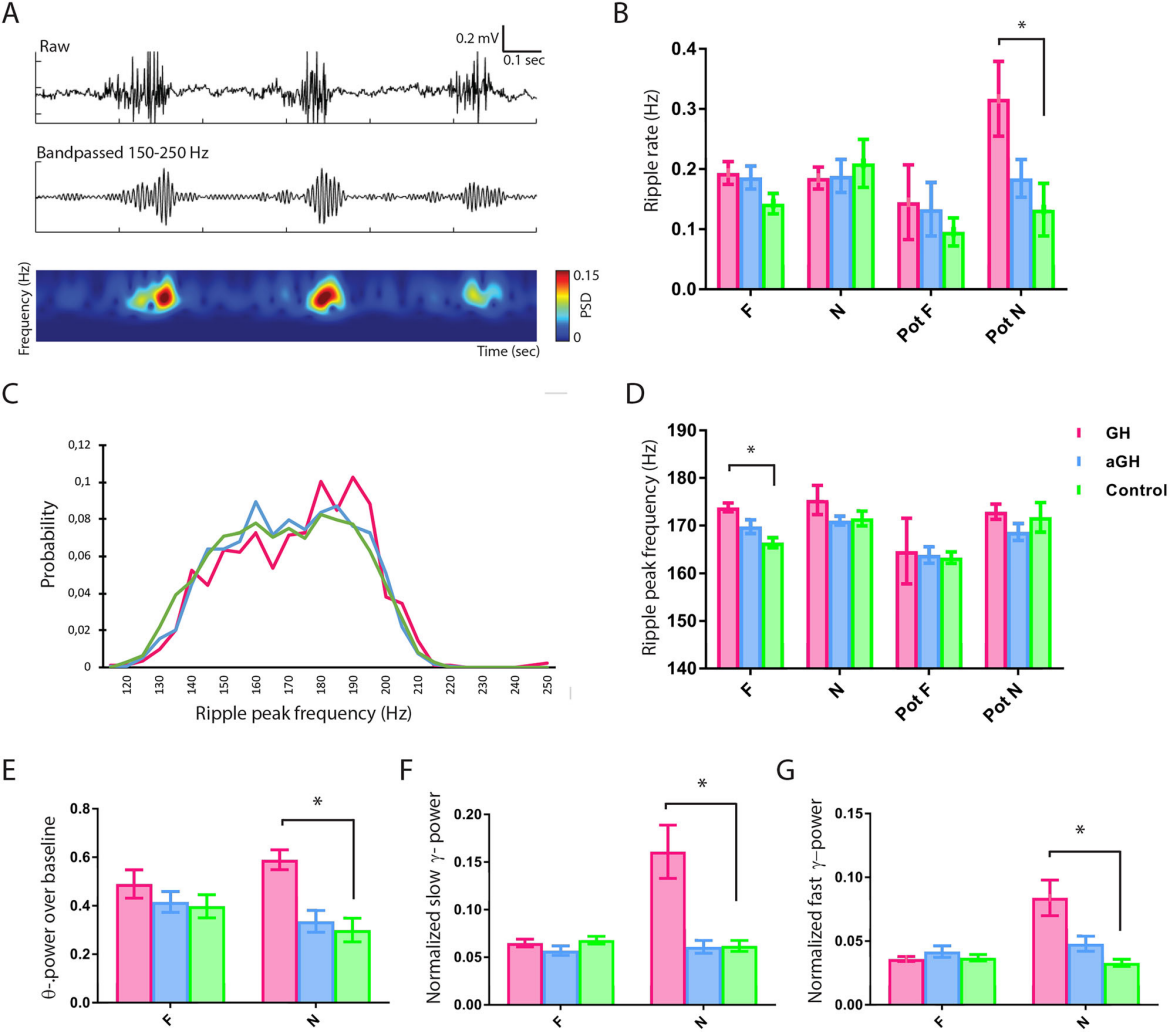
Growth hormone transfection increases ripple occurrence and theta and gamma power in the hippocampal area CA1. ***A***, Top panel, Raw signal of sharp wave ripples (SWRs) from an aGH subject. Middle panel, Signal bandpassed between 150 and 250 Hz. Bottom panel, Spectrogram with color-coded power spectral density (PSD). ***B***, In the pot session after visiting the novel environment (Pot N), GH increased the SWR rate (one-way ANOVA with Bonferroni’s post hoc test, *p* = 0.0392; two-tailed *t* test GH control, *p* = 0.0356) as compared with the control group. ***C***, Probability distribution of SWR peak frequencies. GH increased the probability of higher SWR peak frequencies. ***D***, GH increased the SWR peak frequency significantly at immobile time periods during the exploration of the familiar (F) environment (one-way ANOVA with Bonferroni’s post hoc test, *p* = 0.0043; two-tailed *t* test GH control, *p* < 0.0001). ***E***, The theta power over baseline increased in the GH group during exploration of the novel (N) environment (two-way ANOVA with Bonferroni’s post hoc test, group factor, *p* = 0.0017; two-tailed *t* test GH control, *p* = 0.0012). ***F***, GH increased the normalized slow gamma power during exploration of N (two-way ANOVA with Bonferroni’s post hoc test, group factor, *p* < 0.0001; two-tailed *t* test GH control, *p* = 0.001). ***G***, In addition, GH increased the normalized high gamma power in N (two-way ANOVA with Bonferroni’s post hoc test, group factor, *p* = 0.0003; two-tailed *t* test GH control, *p* = 0.001).

Since GH is naturally secreted during the onset of slow-wave sleep, we hypothesized that GH could affect memory and consolidation by interfering with SWRs. Both SWRs and GH secretion are associated with memory processes in the hippocampus (Li et al., 2011; Buzsaki, 2015), but the association between GH and SWR has never been studied before. We detected SWRs (Fig. 4*A*) in pot sessions and during exploration of the boxes. Only SWRs during immobility were used for the analyses. In the pot session after visiting the novel environment (Pot N), we found that GH increased the SWR rate (Fig. 4*B*; one-way ANOVA with Bonferroni’s post hoc test, *p* = 0.0392; two-tailed *t* test GH control, *p* = 0.0356). In addition, GH increased the SWR peak frequencies during exploration of the familiar environment (Fig. 4*C*,*D*; two-way ANOVA with Bonferroni’s post hoc test, group factor, *p* = 0.0017; two-tailed *t* test GH control, *p* = 0.0012).

Lastly, since theta activity is known to be involved in place cell remapping (Jezek et al., 2011) and theta–gamma cross-frequency coupling is associated with successful pattern separation processes (Zheng et al., 2019), we asked if GH had effects on these oscillations. During exploration of the novel environment, we found that GH increased the theta-power baseline (Fig. 4*E*; two-way ANOVA with Bonferroni’s post hoc test, group factor, *p* = 0.0017; two-tailed *t* test GH control, *p* = 0.0012). GH also increased the normalized slow gamma power (Fig. 4*F*; two-way ANOVA with Bonferroni’s post hoc test, group factor, *p* < 0.0001; two-tailed *t* test GH control, *p* = 0.001), as well as the normalized high gamma power in the novel environment (Fig. 4*G*; two-way ANOVA with Bonferroni’s post hoc test, group factor, *p* = 0.0003; two-tailed *t* test GH control, *p* = 0.001).

## Discussion

Changes in sensory information can update the hippocampal representation of space, and this process depends on intrinsic properties of the network and endocrine modulation. In our study, we report that GH influences the firing properties of CA1 place cells in the hippocampus in several ways. As expected, control animals displayed changes in rate overlap and rate changes when introduced to the novel environment, a phenomenon known as rate remapping. However, the GH cells tended to globally remap instead, as the GH place fields lacked spatial correlation between the familiar and the novel environment. This may indicate that pattern separation was stronger in GH rats. In the familiar environment, reduced GH signaling by the aGH increased the peak rate and bursting of CA1 cells, while the place field size and sparseness decreased. When the aGH animals explored the novel arena in the same room, the place cells maintained high firing rates, with small place field sizes and low sparseness. These results indicate that low levels of GH in CA1 induce rigid and robust hippocampal signaling, not flexible to subtle changes in novel information. Since we have previously demonstrated that GH enhances hippocampal memory by using the same viral transfections (Haugland et al., 2020), we believe that the observed changes in firing rate and spatial correlation are coupled with the changed plasticity in the hippocampus.

The intrinsic plasticity of the hippocampus affects the computation of incoming information to the place cells (Debanne and Poo, 2010; Cobar et al., 2017; Buzsaki and Tingley, 2018). Neuromodulators are perfect candidates to change the neural signaling as they can modulate the spike frequency and synaptic properties, to alter the functional and behavioral outcome (Edelmann et al., 2017; Palacios-Filardo and Mellor, 2019). Although the mechanisms of GH modulations are elusive, the actions of GH are known to be associated with the NMDA receptor (Le Greves et al., 2006; Studzinski et al., 2015). The NMDA receptor is important for long-term stabilization of place cells (McHugh et al., 1996; Kentros et al., 1998), and GH is known to enhance excitatory transmission through NMDA receptors in the CA1 (Molina et al., 2012, 2013). By increasing the plasticity in CA1, GH may increase the sensitivity for new information. Contrarily, enhanced stability is suggested to be associated with a higher peak rate (Cohen et al., 2017), which is what we see in the aGH animals. The altered baseline firing could suggest that the threshold for new sensory inputs is higher. This could also explain why the place cells of the aGH animals kept bursting at an elevated level in the familiar environment. As aGH decreases the spine density in the CA1 (Haugland et al., 2020), increased bursting may be a mechanism for compensating for the reduction of spines. Interestingly, aGH provided increased average firing of the place cells in the novel environment similar to what is reported in aged animals (Wilson et al., 2003, 2005, 2006). We have previously reported memory deficiency of aGH animals in the Morris water maze, which also has been described in aged animals (Gallagher et al., 1993). Since aGH seems to reduce hippocampal plasticity, our findings are in line with a report claiming that low-firing rate place cells are more plastic (Grosmark and Buzsaki, 2016).

When intracranial GH levels are altered, compensatory changes in the systemic secretion of GH by the pituitary glands could take place. However, in our previous study (Haugland et al., 2020) with identical GH manipulation using AAV, we did not observe any motor or body weight changes that would indicate a pituitary role in our study. Further experiments are required to investigate compensatory functions in subjects with low hippocampal GH levels.

The increased firing rate observed in the aGH animals may represent the rigidity of encoding due to failure in the dentate gyrus to reduce the similarity of input patterns sufficiently. In a previous study (Haugland et al., 2020), we reported that aGH animals needed 1 day of extra training to locate a hidden watermaze platform. Upon entry into a novel environment, the balance of incoming sensory and stored information forces the hippocampal network to either settle upon the previously stored representation or create a new representation. In this study, we found that increased levels of GH tended to induce global remapping instead of rate remapping in the novel environment, with only subtle changes. A possible explanation for this could be that GH induces pattern separation processes, which may be a mechanism of enhanced hippocampal-dependent memory functions.

In global remapping, the experience in each environment becomes organized in separate spatial maps such that smaller changes to features within an environment can be detected (Solstad et al., 2014). GH may therefore facilitate pattern separation processes to make the animal better aware of subtle changes in the environment. Moreover, the hippocampus is interconnected with the entorhinal cortex (EC), with interdependencies crucial for functional activity. While the stabilization of place cell representation after global remapping is associated with sensory information from the EC (Miao et al., 2015; Rueckemann et al., 2016; Kanter et al., 2017; Schlesiger et al., 2018), rate remapping in CA1 is proposed to depend on the information originating in the EC layer 3 (Middleton and McHugh, 2016). Since aGH impairs rate remapping this may be due to reduced novelty inputs or increased consolidation processed during the exploration.

Hippocampal gamma oscillations can be defined as slow and fast gamma, in which low gamma is associated with retrieval and fast gamma activity is related to encoding of hippocampal memories (Colgin et al., 2009; Griffiths et al., 2019). GH increased both fast and slow gamma power in our study. Since fast gamma in CA1 and EC is argued to be related to novel learning, this phenomenon could indicate a mechanism for the enhanced remapping of place cells in our study and the improved memory performance by GH (Haugland et al., 2020). On the other hand, studies show that low gamma activity in CA1 is important for correct performance in associative memory tasks (Bieri et al., 2014; Rangel et al., 2016; Zheng et al., 2016). By increasing both fast and slow gamma in CA1, GH might utilize different mechanisms for enhancing pattern separation and learning novel episodic memories.

Memory consolidation processes in the hippocampus are associated with the events of SWR (Buzsaki, 2015). GH is naturally secreted during the onset of slow-wave sleep, the sleep phase that contains SWR, suggesting a role in memory consolidation mechanisms. In our study, we indeed found that GH enhanced SWR rate and peak frequency. This is interesting as SWR declines in rate and peak frequency during normal aging (Wiegand et al., 2016; Cowen et al., 2020). Since the concentration of GH reduces with age, we speculate whether the cognitive decline during aging can be associated with low levels of GH and reduced capacity for consolidation. Taken together, our study shows that GH can modulate hippocampal memory processes and points to the possible mechanism behind the effects. Nevertheless, more studies are required in order to pinpoint the mechanism better.

## Synthesis

Reviewing Editor: Tod E Kippin, University of California Santa Barbara Decisions are customarily a result of the Reviewing Editor and the peer reviewers coming together and discussing their recommendations until a consensus is reached. When revisions are invited, a fact-based synthesis statement explaining their decision and outlining what is needed to prepare a revision will be listed below. The following reviewer(s) agreed to reveal their identity: Todd Green, Lawrence Grover. Note: If this manuscript was transferred from JNeurosci and a decision was made to accept the manuscript without peer review, a brief statement to this effect will instead be what is listed below.

I have received two independent reviews of your manuscript and while both reviewers commented on the strengths of the study, there were concerns that need to be addressed before a final decision regarding suitability for publication can be reached.

Reviewer 1

This manuscript deals with the effect of growth hormone on hippocampal place cells. The experiments were well-done and answered the questions posed by the authors.

The Introduction explains the background to allow the reader to understand why the experiments were done.

The methods are detailed enough for another researcher to follow.

Why only male rats?

I suggest on page 8 to define SWRs in either the title of the section or in line 242.

The results were clear and detailed.

On page 11, line 323, why are there so many fewer GH place cells compared to control and aGH?

While it is described in the Discussion on page 15, it might help the reader to understand the significance of SWRs on page 12 to include 1-2 sentences on the connection between slow wave sleep, GH, and SWRs.

The Discussion summarizes the paper well without over interpreting the data.

On page 13, line 397, there is the sentence that contains "induces a rigid and robust hippocampal,". Is there a word missing here?

On page 15, line 440, define EC.

Reviewer 2

In this manuscript, the authors use molecular techniques to probe potential roles of GH in hippocampal spatial memory functions (stability and plasticity of place fields in familiar and novel environments). This is an intriguing and important experimental issue, given the interrelated roles of GH as a neuromodulator in hippocampal plasticity, the secretion of GH and consolidation of memories during sleep, and the aging associated reduction of GH secretion and memory function. While the present study has considerable potential significance, there are some major problems with the present manuscript.

Major issues

1) In Methods (p.4, line 110) the authors state they only used male rats, but they do not explain why they made this choice. Use of male animals only might be justifiable, but authors need to provide a good rationale.

2) In Results (p. 10, lines 309-310) the authors say "histology revealed that the tetrodes were lowered into the dorsal CA1", but the examples shown in Fig 1A show placement in CA3, not CA1; in addition, the "traces" in Fig 1B are drawn in and the actual electrode track cannot be seen.

3) In Fig 1A, the authors show that their injections were sufficient to cause hippocampal expression of GFP (and therefore GH or aGH), but the micrographs are too high magnification to determine if the expression was specific (limited to) the hippocampus. The authors should show lower magnification images to allow assessment of possible spread of injected virus outside the hippocampus. This is a concern because if the injections spread to sensory cortex or entorhinal cortex, then GH/aGH expression might have altered input to the hippocampus without directly altering hippocampal function. The possibility of injection spread is raised again by results shown in Fig 1C, where whole brain GH was significantly increased by injection. Because GH is expressed endogenously within the brain, I would have expected GH RNA resulting from injections limited to the hippocampus to be obscured by large background signal from expression within the rest of the brain (unless the GH overexpression was extremely strong).

Other issues

1) IGF-I expression should have been measured. IGF-I is also an endogenous neuromodulator with many similar effects to GH, and at least in some tissues, IGF-I expression would be expected to increase with GH overexpression

2) Assay of GH (and IGF-I) protein would be more relevant that RNA.

3) Is there any evidence that the expression level of aGH that was achieved was sufficiently high to block GHR signaling? This is relevant because in the Discussion (p 13, lines 393-394) the authors state that "blockage of the GH receptor by aGH increased the peak rate and bursting of CA1 cells", but they did not provide any evidence that the level of aGH expression was sufficient to block GH receptors.

Minor issues

1) In Methods (p. 7, line 219) is "squire" supposed to be "square"?

2) In the Fig 4B legend (p 22, line 739), the authors state that "In the sleep session after visiting the novel environment (Pot N), GH increased the SWR rate", yet in the Methods (p 8, lines 247-248) and Results (p 12, line 368) the authors says SWRs were recorded during immobility. Animals can be immobile without being asleep. Unless the authors confirmed that the animals were actually asleep, the legend should be changed to indicate immobility (not sleep).

3) In the Discussion (p 15, line 445) the Authors write "Hippocampal gamma oscillations can be defined as low and high low gamma". Did the authors mean "low and high frequency gamma" or "slow and fast gamma"?

4) In the Abstract (p 2, lines 43-44) the Authors write "The increase in firing rate of aGH animals could reflect a compensatory mechanism of the network or the lack of inhibition" yet this suggestion is not followed up later in the manuscript. The authors should either elaborate on this (in the Discussion) or delete this from the Abstract.

5) Some discussion of the relative importance of hippocampal vs pituitary GH would be welcomed. For example, some of the effects the authors observed in GH overexpressing animals may be due to an obscuring of the normal pulsatility of pituitary GH secretion. GH overexpressing animals are likely to have continuously high hippocampal GH levels, compared to normal animals where GH is secreted in pulses. Moreover, since GH can cross the blood-brain barrier, hippocampal GH overexpression might be sufficient to increase circulating GH, thereby affecting peripheral IGF-I production and/or negative feedback of the hypothalamic-pituitary GH/IGF-I axis.
